# Genetic polymorphism association analysis of SNPs on the species conservation genes of Tan sheep and Hu sheep

**DOI:** 10.1007/s11250-019-02063-1

**Published:** 2020-02-05

**Authors:** Hehua EEr, Lina Ma, Xiulan Xie, Jifeng Ma, Xiaoming Ma, Caijuan Yue, Qing Ma, Xiaojun Liang, Wei Ding, Yingkang Li

**Affiliations:** Institute of Animal Science, NingXia Academy of Agriculture and Forestry Sciences, No. 590 Huanghe East Road, Yinchuan, 750002 Ningxia Hui Autonomous Region China

**Keywords:** Tan sheep, SNPs, Association analysis, Biomarker

## Abstract

For further understanding the genetic control mechanisms of growth and development in Tan sheep, and culturing good traits on meat performance, which is very important to both in developing local species and improving economic efficaciously. In our study, we recruited a total of 250 Tan sheep and 174 healthy Hu sheep to detect 32 SNPs in GH, GHR, NPY, Leptin, H-FABP, MSTN, and CAST by using direct sequencing techniques, in order to explore genetic marking loci which were an association with growth characters. From the results, we found different SNPs with an obvious difference for the growth traits. In the different genetic model analysis, we found SNP12, SNP29, SNP41, SNP8, SNP34, SNP35, SNP9, SNP10, SNP36, SNP45, and SNP39 were a significantly negative association with the two kinds of sheep. And SNP46, SNP42, and SNP69 with the positive association between the different trait in sheep were analyzed. From the LD and haplotype analysis, we found three blocks with the positive association in growth traits between Tan sheep and Hu sheep. The block of SNP29, SNP32, SNP34, SNP35, SNP36, SNP39, SNP41, SNP42, SNP45, and SNP46 with the genotype “AATCTACTTA” is the most significantly association with the traits. In summary, the study initially explored the genes for growth and reproduction between Tan sheep and Hu sheep and found some statistically significant results which demonstrate that there are genetic differences. These differential molecular markers may provide a scientific theoretical basis for the preferred species of Tan sheep which with good meat performance and better utilization of species resources.

**Electronic supplementary material:**

The online version of this article (10.1007/s11250-019-02063-1) contains supplementary material, which is available to authorized users.

## Introduction

As an important part of the animal husbandry industry, the sheep industry not only produces raw materials for the wool spinning industry, but also provides people with delicious lamb. Lamb is prevalent in the domestic and international markets because of its characteristics of lean meat, less fat, fresh, juicy, and easy to digest. China’s mutton sheep production needs better varieties with high meat production and good meat quality which require the continuous selection of meat quality in the breeding of sheep to improve the quality of mutton (Zhang et al. 2015).

Tan sheep are a special breed of sheep with excellent suede formed through long-term natural and artificial selection, and artificial breeding (Lv et al. 2009). Tan sheep have the characteristics of drought resistance, salt and alkali resistance, and resistance to roughage, and show great adaptability to desert, semi-desert, and arid steppe (Kang et al. 2013). The character of Tan sheep suede is a combination of light, thin, soft, warm, beautiful, and pleasing (Xu et al. 2011) which listed as one of the “Five Treasures” in Ningxia. As a unique product of Ningxia, Tan sheep is the result of the unique ecological environment in Ningxia (Xu et al. 2015) and listed as the second-tier protected animal by Chinese government (Tao et al. 2017). Grassland in Ningxia is characterized by drought, sparse rain, and low grass production. However, it has the characteristics of high dry matter content, abundant salt, and mineral content, and it is a natural gift for Tan sheep breeding. Hu sheep is one of the important livestock in Taihu Plain of China which has high reproductive rate, long estrus period, and an average litter size of 2.06 (Feng et al. 2018). It not only has high reproductive performance, fast growth performance, and strong environmental adaptability, but also has the advantages of multiple lambs per fetus, good lactation performance, and fast growth and development (Zhicheng et al. 2018).

Intramuscular fat (IMF) content is a very important indicator of meat quality, mainly in the epicardium and muscle fascia as well as the endomysium, which is an important economic trait with moderately high heritability (Hovenier et al. 1993; Won et al. 2018). With the improvement of people’s living standards and dietary structure, people’s demand for mutton consumption continues to enhance (Sahin et al. 2014). However, traditional breeding methods mainly emphasize the improvement of the sheep’s production performance (XC et al. 2015), and the results will lead to a decline in the quality of the lamb meat, especially the meat flavor (Xiong et al. 2017). Chinese mutton sheep production needs better varieties with high meat production and good meat quality that require the continuous selection of meat quality in the breeding of sheep to improve the quality of mutton. Classic breeding methods are mainly through cross breeding, which leaves individuals with excellent quality and breeds an excellent and stable phenotype after several generations of breeding (Bittante et al. 1996). However, the conventional method has great difficulty in selecting meat quality traits. Not only it needs a considerable expense for breeding, but also it cannot be accurately detected.

The formation of meaty traits is essentially the product of the interaction between genes and the environment (Lebret et al. 2015). Studies have shown that genetic factors play a key role in meat quality. In general, 10~30% of the variation of meat quality traits and the quality of meat products are determined by the genetic material of the animal (Lefaucheur 2010; Davoli and Braglia 2007). With the rapid development of molecular biology theory and technology, molecular genetics and genetic engineering methods, combined with the traditional methods of marker-assisted selection, have provided better solutions for the breeding problems (Jamshidi et al. 2009).

As a famous suede sheep breed in China (Cui et al. 1962), Tan sheep are known for the world with its delicious meat. Hu sheep are known with the famous multiplicity sheep varieties in the world. Tan sheep have some prominent problems, such as slow growth and development, long feeding cycle, low reproduction rate, poor breeding efficiency, decline of breeds, and degradation of quality, which are relative advantages of Hu sheep. In view of the intrinsic interaction mechanism and biological functions of *GH* (growth hormone), *GHR* (growth hormone receptor), *NPY* (neuropeptide Y), *Leptin*, *H-FABP* (Heart fatty acid-binding proteins), *MSTN* (myostatin), and *CAST* (calpastatin), the study focused on the genetic effects of their polymorphisms on the growth and development of Tan sheep, with a view to discovering genetic markers with significant effects on growth traits and providing scientific basis for the high-quality breeding of Tan sheep and the protection and utilization of germplasm resources.

## Methods

### Study participants

The study samples include 250 healthy Tan sheep as the case and 174 healthy Hu sheep as the control group (Ningxia Lingwu Luyuan Agriculture and Animal Husbandry Co., Ltd.) to explore the difference. Tan sheep and Hu sheep are all raised in the Ningxia Yanchi County Tanyang Breeding Center. They are in the same ecological environment and are kept in a normally raised according to the sheep farm diet formula. Their recruitment time is from September 2015 to January 2017, and is carried out by means of whole house feeding. The lambs born in the same period were selected to measure their body weight and body size indicators. Body size indicators include body height, body length, and chest circumference. The lamb’s birth weight and body size indicators are measured before the lamb is born and eating colostrum. The selected samples kept the same husbandry and management level, and individual nutritional status in the same period. When they were born 1 year later, we took blood samples from each sheep and labeled them, and then returned to the laboratory at low temperature − 20 °C. According to the animal husbandry methods, we measured the basic growth and development trait indicators which included birth weight, weaning weight, 3 months weight, and 6 months weight which ensure that all sample nutrition levels differ by less than 5%. The animal ethics and welfare committee of NingXia Academy of Agriculture and Forestry Sciences approved the experimental procedures which is in compliance with the regulations for protection of animal research.

### SNP selection and genotyping

For the association analysis, we selected these 32 validated SNPs (single-nucleotide polymorphisms) from some researches (the sequence information listed in supplement--SNPs information). The SNPs (single-nucleotide polymorphism) for the present study were selected following three criteria: (a) the SNP call rate was > 90%, (b) the minor allele frequency (MAF) was > 0.05%, and (c) the allele frequencies of the control group were consistent with the Hardy-Weinberg equilibrium. These selected SNPs have not been named yet and are just some novel SNPs. They are only located in the meat quality–related gene sequences of GH (growth hormone), GHR (growth hormone receptor), NPY (Neuropeptide Y), Leptin, H-FABP (heart fatty acid-binding proteins), MSTN (myostatin), and CAST (calpastatin) according to the Genbank published (listed in the supplement material-SNPs information). DNA was extracted from blood samples using a genomic DNA purification kit (GoldMag, China), and the blood was stored with a condition of − 20 °C. The DNA concentration was measured by spectrometry (DU530 UV/VIS spectrophotometer, Beckman Instruments, Fullerton, CA, USA). The Sequenom MassARRAY Assay Design 4.0 software (Sequenom, Inc., San Diego, CA, USA) was used to design the Multiplexed SNP Mass EXTEND assay (Trembizki et al. 2014). Genotyping SNPs were performed using a Sequenom MassARRAY RS1000 (Sequenom, Inc.) according to the standard protocol.

### Statistical analysis

The SequenomTyper 4.0 Software™ (Sequenom, Inc.) was used to manage and analyze the data. The univariate and multivariable logistic regression models were used to examine the association between Tan sheep and Hu sheep, and corresponding odds ratios (ORs) and 95% confidence intervals (CIs) were calculated simultaneously. All statistical analysis was carried out using SPSS19.0 statistical software (SPSS. Chicago, IL) and Microsoft Excel, and a two-tailed *p* value of < 0.05 was considered statistically significant. Logistic regression analysis was performed on all SNP loci. The analysis models include dominant, recessive, log-additive, and codominant model (which reference to normality homozygous). PLINK software (http://pngu.mgh.harvard.edu/purcell/plink/) was used by the models to evaluate the association of 32 SNPs between the two kinds of sheep. Lastly, Haploview software (version 4.2) was conducted to analyses linkage disequilibrium (LD).

## Results

### Significant SNPs in Tan sheep

Thirty-two SNPs were successfully genotyped in the sheep, and all of the tested SNPs were in accordance with Hardy-Weinberg equilibrium (HWE) in both groups (*p* > 0.01). We detected the minor allele of each SNP which was assumed to be the wild-type allele. MAF (Minor Allele Frequency) in cases and controls was showed in Table 1. Under the allele genetic model through the *χ*^2^ test, we found the SNP12 (OR = 0.56, *p* = 0.000096), SNP29 (OR = 0.55, *p* = 0.000076), SNP41 (OR = 0.57, *p* = 0.0047), SNP8 (OR = 0.199, *p* = 2.3 × 10^−8^), SNP34 (OR = 0.54, *p* = 0.000057), SNP35 (OR = 0.53, *p* = 0.000039), SNP9 (OR = 0.194, *p* = 1.172 × 10^−8^), SNP10 (OR = 0.47, *p* = 0.000011), SNP36(OR = 0.53, *p* = 0.000039), SNP45 (OR = 0.54, *p* = 0.000057), and SNP39 (OR = 0.55, *p* = 0.000076) were significantly negatively correlated with growth trait differences between the two kinds of sheep. And SNP46 (OR = 4.848, *p* = 0.00035), SNP42 (OR = 5.027, *p* = 0.000039), and SNP69 (OR = 1.784, *p* = 0.000083) were positively correlated with these characters. These differences are consistent before and after FDR correction except the SNP61. The association of SNP61 and the trait is OR = 2.456, *p* < 0.05, after FDR, *p* > 0.05. After the *χ*^2^ test in other methods such as codominant, dominant, and recessive, we further analyzed the differences between the two kinds of sheep and found SNP12, SNP29, SNP34, SNP35, SNP9, SNP10, SNP36, SNP45, SNP39, and SNP69 as significant association with the characters of the two groups (Table 2). From the results, we found only the SNP69 has significant significance in all models which means SNP69 would significantly increase the difference of growth traits between Tan sheep and Hu sheep. SNP61 increased the difference at dominant and additive models (OR = 2.534, *p* = 0.035) before FDR correction. In the dominant and log-additive models, SNP46 and SNP42 increased the growth traits difference whether or not FDR corrected. And when SNP46 is the genotype “G/A” and SNP42 with “C/T” in codominant model, we discovered the difference is increased before FDR (false discovery rate) correction. However, SNP12, SNP29, SNP34, SNP35, SNP10, SNP36, SNP45, and SNP39 in these models, all of which, indicated their regulated trait significant difference between the two kinds of sheep (Table 3 and supplement Excel-Table 3).

**Table 1:** The basic characters of 32 SNPs located in 7 genes

SNP	Gene	A	B	HWE	Tan sheep	MAF-Tan	HWE_Tan	Hu sheep	MAF-Hu	HWE_Hu
SNP3	GH	T	C	0.2568	8/84/158	0.2	0.5536	8/70/96	0.2471	0.4136
SNP4	GH	G	A	0.2568	8/84/158	0.2	0.5536	8/70/96	0.2471	0.4136
SNP8	GH	C	T	1	0/14/236	0.028	1	2/40/132	0.1264	1
SNP9	GH	T	A	0.4425	0/14/236	0.028	1	3/39/132	0.1293	1
SNP10	GHR	G	A	0.1245	7/60/183	0.148	0.4501	15/64/95	0.2701	0.4405
SNP12	GHR	C	T	0.5809	20/96/134	0.272	0.6329	27/85/62	0.3994	0.8752
SNP15	NPY	A	G	1	5/55/190	0.13	0.5819	2/41/131	0.1293	0.7428
SNP18	NPY	G	A	0.8032	19/100/131	0.276	1	12/63/99	0.25	0.6858
SNP20	NPY	A	T	1	2/40/208	0.088	1	2/39/133	0.1236	1
SNP21	LEPTIN	A	G	0.1999	0/24/226	0.048	1	3/22/149	0.0805	0.08154
SNP22	LEPTIN	G	A	1	0/4/246	0.008	1	0/0/174	0	1
SNP24	LEPTIN	C	0	1	0/0/248	0	1	0/0/174	0	1
SNP25	LEPTIN	G	A	0.04198	1/7/242	0.018	0.07063	0/0/174	0	1
SNP28	LEPTIN	G	A	0.3478	1/14/235	0.032	0.2194	0/11/163	0.0316	1
SNP29	H-FABP	A	G	0.9071	15/91/144	0.242	0.8643	22/84/68	0.3678	0.7443
SNP32	H-FABP	A	G	1	0/3/247	0.006	1	0/0/174	0	1
SNP34	H-FABP	T	C	0.9065	15/90/145	0.24	0.8623	22/84/68	0.3678	0.7443
SNP35	H-FABP	C	G	0.7262	15/90/145	0.24	0.8623	23/83/68	0.3707	0.8712
SNP36	H-FABP	T	A	0.7262	15/90/145	0.24	0.8623	23/83/68	0.3707	0.8712
SNP39	H-FABP	A	G	0.9071	15/91/144	0.242	0.8643	22/84/68	0.3678	0.7443
SNP41	H-FABP	C	T	1	12/76/162	0.2	0.4299	13/80/81	0.3046	0.3697
SNP42	H-FABP	C	T	0.6007	1/32/216	0.0683	1	0/5/169	0.0144	1
SNP45	H-FABP	T	C	0.9065	15/90/145	0.24	0.8623	22/84/68	0.3678	0.7443
SNP46	H-FABP	G	A	0.5801	1/31/218	0.066	1	0/5/169	0.0144	1
SNP47	MSTN	A	0	1	0/0/250	0	1	0/0/174	0	1
SNP48	MSTN	A	0	1	0/0/250	0	1	0/0/174	0	1
SNP50	MSTN	A	0	1	0/0/250	0	1	0/0/174	0	1
SNP51	MSTN	T	0	1	0/0/250	0	1	0/0/174	0	1
SNP54	MSTN	A	0	1	0/0/249	0	1	0/0/174	0	1
SNP57	MSTN	A	0	1	0/0/250	0	1	0/0/174	0	1
SNP61	MSTN	T	C	1	0/24/226	0.048	1	0/7/167	0.0201	1
SNP69	CAST	A	G	0.6796	46/124/80	0.432	0.8982	12/80/82	0.2989	0.2767

*HWE*, Hardy-Weinberg equilibrium; *MAF*, minor allele frequency

**Table 2:** The significantly results about 32 SNPs in genetic models between two kinds of sheep

SNP	Model	Genotype	Tan	Hu	ChiScore	OR (95% CI)	*p*	FDR_BH adjusted^b^
SNP8	Codominant	C/C	236	132	31.29		1.60E−07^a^	
		C/T	14	40				
		T/T	0	2				
	Dominant	C/C	236	132	30.76		2.93E−08	
		C/T-T/T	14	42				
	Recessive	C/C-C/T	250	172	2.887		8.93E−02	
		T/T	0	2				
	Allele	C	486	304	31.2	0.199 (0.1073–0.3693)	2.32E−08	5.58E−07
		T	14	44				
SNP9	Codominant	T/T	236	132	31.58		1.39E−07	
		T/A	14	39				
		A/A	0	3				
	Dominant	T/T	236	132	30.76		2.93E−08	
		T/A-A/A	14	42				
	Recessive	T/T-T/A	250	171	4.341		3.72E−02	
		A/A	0	3				
	Allele	T	486	303	32.53	0.194 (0.1047–0.3594)	1.17E−08	2.93E−07
		A	14	45				
SNP10	Codominant	G/G	183	95	17.84		1.33E−04	
		G/A	60	64				
		A/A	7	15				
	Dominant	G/G	183	95	15.73		7.33E−05	
		G/A-A/A	67	79				
	Recessive	G/G-G/A	243	159	7.066		7.86E−03	
		A/A	7	15				
	Allele	G	426	254	19.26	0.4694 (0.3334–0.6607)	1.14E−05	2.62E−04
		A	74	94				
SNP12	Codominant	C/C	134	62	15.02		5.48E−04	
		C/T	96	85				
		T/T	20	27				
	Dominant	C/C	134	62	13.32		2.62E−04	
		C/T-T/T	116	112				
	Recessive	C/C-C/T	230	147	5.882		1.53E−02	
		T/T	20	27				
	Allele	C	364	209	15.2	0.5618 (0.4198–0.7517)	9.65E−05	1.45E−03
		T	136	139				
SNP21	Codominant	A/A	226	149	5.45		6.55E−02	
		A/G	24	22				
		G/G	0	3				
	Dominant	A/A	226	149	2.282		1.31E−01	
		A/G-G/G	24	25				
	Recessive	A/A-A/G	250	171	4.341		3.72E−02	
		G/G	0	3				
	Allele	A	476	320	3.756	0.5762 (0.3281–1.012)	5.26E−02	3.85E−01
		G	24	28				
SNP25	Codominant	G/G	242	174	5.675		5.86E−02	
		G/A	7	0				
		A/A	1	0				
	Dominant	G/G	242	174	5.675		1.72E−02	
		G/A-A/A	8	0				
	Recessive	G/G-G/A	249	174	0.6976		4.04E-01	
		A/A	1	0				
	Allele	G	491	348	6.331	NA (NA–NA)	1.19E−02	1.23E−01
		A	9	0				
SNP29	Codominant	A/A	144	68	15.73		3.84E−04	
		A/G	91	84				
		G/G	15	22				
	Dominant	A/A	144	68	14.07		1.76E−04	
		A/G-G/G	106	106				
	Recessive	A/A-A/G	235	152	5.685		1.71E−02	
		G/G	15	22				
	Allele	A	379	220	15.66	0.5487 (0.4069–0.7399)	7.58E−05	1.36E−03
		G	121	128				
SNP34	Codominant	T/T	145	68	16.27		2.94E−04	
		T/C	90	84				
		C/C	15	22				
	Dominant	T/T	145	68	14.69		1.27E−04	
		T/C-C/C	105	106				
	Recessive	T/T-T/C	235	152	5.685		1.71E−02	
		C/C	15	22				
	Allele	T	380	220	16.2	0.5428 (0.4024–0.7322)	5.70E−05	1.14E−03
		C	120	128				
SNP35	Codominant	C/C	145	68	16.72		2.34E−04	
		C/G	90	83				
		G/G	15	23				
	Dominant	C/C	145	68	14.69		1.27E−04	
		C/G-G/G	105	106				
	Recessive	C/C-C/G	235	151	6.552		1.05E−02	
		G/G	15	23				
	Allele	C	380	219	16.9	0.5361 (0.3975–0.723)	3.95E−05	8.68E−04
		G	120	129				
SNP36	Codominant	T/T	145	68	16.72		2.34E−04	
		T/A	90	83				
		A/A	15	23				
	Dominant	T/T	145	68	14.69		1.27E−04	
		T/A-A/A	105	106				
	Recessive	T/T-T/A	235	151	6.552		1.05E−02	
		A/A	15	23				
	Allele	T	380	219	16.9	0.5361 (0.3975–0.723)	3.95E−05	8.68E−04
		A	120	129				
SNP39	Codominant	A/A	144	68	15.73		3.84E−04	
		A/G	91	84				
		G/G	15	22				
	Dominant	A/A	144	68	14.07		1.76E−04	
		A/G-G/G	106	106				
	Recessive	A/A-A/G	235	152	5.685		1.71E−02	
		G/G	15	22				
	Allele	A	379	220	15.66	0.5487 (0.4069–0.7399)	7.58E−05	1.36E−03
		G	121	128				
SNP41	Codominant	C/C	162	81	13.97		9.26E−04	
		C/T	76	80				
		T/T	12	13				
	Dominant	C/C	162	81	13.96		1.86E−04	
		C/T-T/T	88	93				
	Recessive	C/C-C/T	238	161	1.319		2.51E−01	
		T/T	12	13				
	Allele	C	400	242	12.21	0.5708 (0.4159–0.7832)	4.76E−04	5.70E−03
		T	100	106				
SNP42	Codominant	C/C	216	169	13.57		1.13E−03	
		C/T	32	5				
		T/T	1	0				
	Dominant	C/C	216	169	13.5		2.39E−04	
		C/T-T/T	33	5				
	Recessive	C/C-C/T	248	174	0.7005		4.03E−01	
		T/T	1	0				
	Allele	C	464	343	13.54	5.027 (1.946–12.99)	2.34E−04	3.27E−03
		T	34	5				
SNP45	Codominant	T/T	145	68	16.27		2.94E−04	
		T/C	90	84				
		C/C	15	22				
	Dominant	T/T	145	68	14.69		1.27E−04	
		T/C-C/C	105	106				
	Recessive	T/T-T/C	235	152	5.685		1.71E−02	
		C/C	15	22				
	Allele	T	380	220	16.2	0.5428 (0.4024–0.7322)	5.70E−05	1.14E-03
		C	120	128				
SNP46	Codominant	G/G	218	169	12.77		1.69E−03	
		G/A	31	5				
		A/A	1	0				
	Dominant	G/G	218	169	12.69		3.67E−04	
		G/A-A/A	32	5				
	Recessive	G/G-G/A	249	174	0.6976		4.04E−01	
		A/A	1	0				
	Allele	G	467	343	12.78	4.848 (1.873–12.55)	3.50E−04	4.55E-03
		A	33	5				
SNP61	Codominant	T/T	226	167	NA		NA	
		T/C	24	7				
		C/C	0	0				
	Dominant	T/T	226	167	4.709		3.00E−02	
		T/C-C/C	24	7				
	Recessive	T/T-T/C	250	174	NA		NA	
		C/C	0	0				
	Allele	T	476	341	4.53	2.456 (1.046–5.766)	3.33E−02	2.87E−01
		C	24	7				
		A	16	11				
SNP69	Codominant	A/A	80	82	16.35		2.82E−04	
		A/G	124	80				
		G/G	46	12				
	Dominant	A/A	80	82	9.943		1.62E−03	
		A/G-G/G	170	92				
	Recessive	A/A-A/G	204	162	11.5		6.97E−04	
		G/G	46	12				
	Allele	A	284	244	15.48	1.784 (1.335–2.384)	8.33E−05	1.36E−03
		G	216	104				

^a^Indicate that the data is statistically significant (*p* < 0.05)

^b^FDR_BH: false discovery rate_ Benjamini-Hochberg correction

**Table 3:** The significant results of logistic regression analysis within all SNP loci

SNP	Model	Genotype	Tan	Hu	OR (95% CI)	*P* value	FDR_BH adjusted
SNP8	Codominant	C/C	236	132	–	–	–
		C/T	14	40	0.1958 (0.1027–0.3731)	*7.16E−07*^*a*^	1.00E+00
		T/T	0	2	3.462e−10 (0-inf)	9.99E−01	1.00E+00
	Dominant	C/C	236	132	0.1864 (0.09818–0.354)	*2.84E−07*^*a*^	*4.55E−06*^*b*^
		C/T-T/T	14	42			
	Recessive	C/C-C/T	250	172	4.259e−10 (0-inf)	9.99E−01	1.00E+00
		T/T	0	2			
	Additive	–	–	–	0.1909 (0.1013–0.3599)	*3.07E−07*^*a*^	*5.04E−06*^*b*^
SNP9	Codominant	T/T	236	132	–	–	–
		T/A	14	39	0.2008 (0.1052–0.3834)	*1.14E−06*^*a*^	1.00E+00
		A/A	0	3	3.462e−10 (0-inf)	9.99E−01	1.00E+00
	Dominant	T/T	236	132	0.1864 (0.09818–0.354)	*2.84E−07*^*a*^	*4.55E−06*^*b*^
		T/A-A/A	14	42			
	Recessive	T/T-T/A	250	171	4.234e−10 (0-inf)	9.99E−01	1.00E+00
		A/A	0	3			
	Additive	–	–	–	0.1931 (0.1028–0.3627)	*3.15E−07*^*a*^	*5.04E−06*^*b*^
SNP10	Codominant	G/G	183	95	–	–	–
		G/A	60	64	0.4867 (0.3164–0.7487)	*1.05E−03*^*a*^	*1.01E−02*^*b*^
		A/A	7	15	0.2423 (0.09551–0.6145)	*2.83E−03*^*a*^	*1.01E−02*^*b*^
	Dominant	G/G	183	95	0.4403 (0.2924–0.6628)	*8.50E−05*^*a*^	*6.43E−04*^*b*^
		G/A-A/A	67	79			
	Recessive	G/G-G/A	243	159	0.3053 (0.1218–0.7656)	*1.14E−02*^*a*^	6.95E−02
		A/A	7	15			
	Additive	–	–	–	0.4892 (0.3483–0.687)	*3.69E−05*^*a*^	*3.25E−04*^*b*^
SNP12	Codominant	C/C	134	62	–	–	–
		C/T	96	85	0.5226 (0.3436–0.7948)	*2.42E−03*^*a*^	*7.78E−03*^*b*^
		T/T	20	27	0.3427 (0.1786–0.6578)	*1.28E−03*^*a*^	*7.78E−03*^*b*^
	Dominant	C/C	134	62	0.4792 (0.322–0.7131)	*2.87E−04*^*a*^	*8.35E−04*^*b*^
		C/T-T/T	116	112			
	Recessive	C/C-C/T	230	147	0.4734 (0.2562–0.8749)	*1.70E−02*^*a*^	6.95E−02
		T/T	20	27			
	Additive	–	–	–	0.5645 (0.4201–0.7583)	*1.47E−04*^*a*^	*4.27E−04*^*b*^
SNP29	Codominant	A/A	144	68	–	–	–
		A/G	91	84	0.5116 (0.3383–0.7737)	*1.49E−03*^*a*^	*7.78E−03*^*b*^
		G/G	15	22	0.322 (0.1572–0.6594)	*1.95E−03*^*a*^	*7.78E−03*^*b*^
	Dominant	A/A	144	68	0.4722 (0.3183–0.7006)	*1.93E−04*^*a*^	*6.56E−04*^*b*^
		A/G-G/G	106	106			
	Recessive	A/A-A/G	235	152	0.441 (0.2218–0.8768)	*1.96E−02*^*a*^	6.95E−02
		G/G	15	22			
	Additive	–	–	–	0.5441 (0.4003–0.7396)	*1.02E−04*^*a*^	*3.25E−04*^*b*^
SNP34	Codominant	T/T	145	68	–	–	–
		T/C	90	84	0.5025 (0.3321–0.7602)	*1.12E−03*^*a*^	*7.78E−03*^*b*^
		C/C	15	22	0.3197 (0.1561–0.6548)	*1.82E−03*^*a*^	*7.78E−03*^*b*^
	Dominant	T/T	145	68	0.4645(0.313–0.6894)	*1.41E−04*^*a*^	*6.43E−04*^*b*^
		T/C-C/C	105	106			
	Recessive	T/T-T/C	235	152	0.441 (0.2218–0.8768)	*1.96E−02*^*a*^	6.95E*−*02
		C/C	15	22			
	Additive	–	–	–	0.5391 (0.3965–0.7328)	*7.99E−05*^*a*^	*3.25E−04*^*b*^
SNP35	Codominant	C/C	145	68	–	–	–
		C/G	90	83	0.5085 (0.3359–0.7698)	*1.39E−03*^*a*^	*7.78E−03*^*b*^
		G/G	15	23	0.3058 (0.1502–0.623)	*1.10E−03*^*a*^	*7.78E−03*^*b*^
	Dominant	C/C	145	68	0.4645 (0.313–0.6894)	*1.41E−04*^*a*^	*6.43E−04*^*b*^
		C/G-G/G	105	106			
	Recessive	C/C-C/G	235	151	0.4191 (0.2119–0.8287)	*1.24E−02*^*a*^	6.95E−02
		G/G	15	23			
	Additive	–	–	–	0.5349 (0.394–0.7262)	*6.06E−05*^*a*^	*3.25E−04*^*b*^
SNP36	Codominant	T/T	145	68	–	–	–
		T/A	90	83	0.5085 (0.3359–0.7698)	*1.39E−03*^*a*^	*7.78E−03*^*b*^
		A/A	15	23	0.3058 (0.1502–0.623)	*1.10E−03*^*a*^	*7.78E−03*^*b*^
	Dominant	T/T	145	68	0.4645 (0.313–0.6894)	*1.41E−04*^*a*^	*6.43E−04*^*b*^
		T/A-A/A	105	106			
	Recessive	T/T-T/A	235	151	0.4191 (0.2119–0.8287)	*1.24E−02*^*a*^	6.95E−02
		A/A	15	23			
	Additive	–	–	–	0.5349 (0.394–0.7262)	*6.06E−05*^*a*^	*3.25E−04*^*b*^
SNP39	Codominant	A/A	144	68	–	–	–
		A/G	91	84	0.5116 (0.3383–0.7737)	*1.49E−03*^*a*^	*7.78E−03*^*b*^
		G/G	15	22	0.322 (0.1572–0.6594)	*1.95E−03*^*a*^	*7.78E−03*^*b*^
	Dominant	A/A	144	68	0.4722 (0.3183–0.7006)	*1.93E−04*^*a*^	*6.56E−04*^*b*^
		A/G-G/G	106	106			
	Recessive	A/A-A/G	235	152	0.441 (0.2218–0.8768)	*1.96E−02*^*a*^	6.95E−02
		G/G	15	22			
	Additive	–	–	–	0.5441 (0.4003–0.7396)	*1.02E−04*^*a*^	*3.25E−04*^*b*^
SNP41	Codominant	C/C	162	81	–	–	–
		C/T	76	80	0.475 (0.3146–0.7171)	*3.97E−04*^*a*^	2.16E−01
		T/T	12	13	0.4615 (0.2015–1.057)	6.75E−02	2.16E−01
	Dominant	C/C	162	81	0.4731 (0.3187–0.7024)	*2.05E−04*^*a*^	*6.56E−04*^*b*^
		C/T-T/T	88	93			
	Recessive	C/C-C/T	238	161	0.6244 (0.2779–1.403)	2.54E−01	8.14E−01
		T/T	12	13			
	Additive	–	–	–	0.5659 (0.4094–0.7822)	*5.65E−04*^*a*^	*1.51E−03*^*b*^
SNP42	Codominant	C/C	216	169	–	–	–
		C/T	32	5	5.007 (1.91–13.13)	*1.05E−03*^*a*^	1.00E+00
		T/T	1	0	1.264e+09 (0-inf)	9.99E−01	1.00E+00
	Dominant	C/C	216	169	5.164 (1.974–13.51)	*8.22E−04*^*a*^	*2.19E−03*^*b*^
		C/T-T/T	33	5			
	Recessive	C/C-C/T	248	174	1.133e+09 (0-inf)	9.99E−01	1.00E+00
		T/T	1	0			
	Additive	–	–	–	5.078 (1.953–13.21)	*8.61E−04*^*a*^	*2.12E−03*^*b*^
SNP45	Codominant	T/T	145	68	–	–	–
		T/C	90	84	0.5025 (0.3321–0.7602)	*1.12E−03*^*a*^	*7.78E−03*^*b*^
		C/C	15	22	0.3197 (0.1561–0.6548)	*1.82E−03*^*a*^	*7.78E−03*^*b*^
	Dominant	T/T	145	68	0.4645 (0.313–0.6894)	*1.41E−04*^*a*^	*6.43E−04*^*b*^
		T/C-C/C	105	106			
	Recessive	T/T-T/C	235	152	0.441 (0.2218–0.8768)	*1.96E−02*^*a*^	6.95E−02
		C/C	15	22			
	Additive	–	–	–	0.5391 (0.3965–0.7328)	*7.99E−05*^*a*^	*3.25E−04*^*b*^
SNP46	Codominant	G/G	218	169	–	–	–
		G/A	31	5	4.806 (1.83–12.62)	*1.44E−03*^*a*^	1.00E+00
		A/A	1	0	1.252e+09 (0-inf)	9.99E−01	1.00E+00
	Dominant	G/G	218	169	4.961 (1.893–13.01)	*1.12E−03*^*a*^	*2.77E−03*^*b*^
		G/A-A/A	32	5			
	Recessive	G/G-G/A	249	174	1.129e+09 (0-inf)	9.99E−01	1.00E+00
		A/A	1	0			
	Additive	–	–	–	4.879 (1.874–12.71)	*1.17E−03*^*a*^	*2.67E−03*^*b*^
SNP61	Codominant	T/T	226	167	–	–	–
		T/C	24	7	NA (NA–NA)	NA	1.00E+00
		C/C	0	0	NA (NA–NA)	NA	1.00E+00
	Dominant	T/T	226	167	2.534 (1.066–6.019)	*3.53E−02*^*a*^	7.52E−02
		T/C-C/C	24	7			
	Recessive	T/T-T/C	250	174	NA (NA–NA)	NA	1.00E+00
		C/C	0	0			
	Additive	–	–	–	2.534 (1.066–6.019)	*3.53E−02*^*a*^	7.52E−02
SNP69	Codominant	A/A	80	82	–	–	–
		A/G	124	80	1.589 (1.047–2.411)	*2.96E−02*^*a*^	*4.65E−03*^*b*^
		G/G	46	12	3.929 (1.939–7.96)	*1.45E−04*^*a*^	*4.65E−03*^*b*^
	Dominant	A/A	80	82	1.894 (1.271–2.823)	*1.71E−03*^*a*^	*3.90E−03*^*b*^
		A/G-G/G	170	92			
	Recessive	A/A-A/G	204	162	3.044 (1.561–5.937)	*1.09E−03*^*a*^	*3.49E−02*^*b*^
		G/G	46	12			
	Additive	–	–	–	1.831 (1.354–2.476)	*8.59E−05*^*a*^	*3.25E−04*^*b*^

^a^Indicates that the data is statistically significant when before adjustment

^b^Indicates that the data is statistically significant when after FDR-BH correction

### Haplotype analysis

**Fig. 1:**
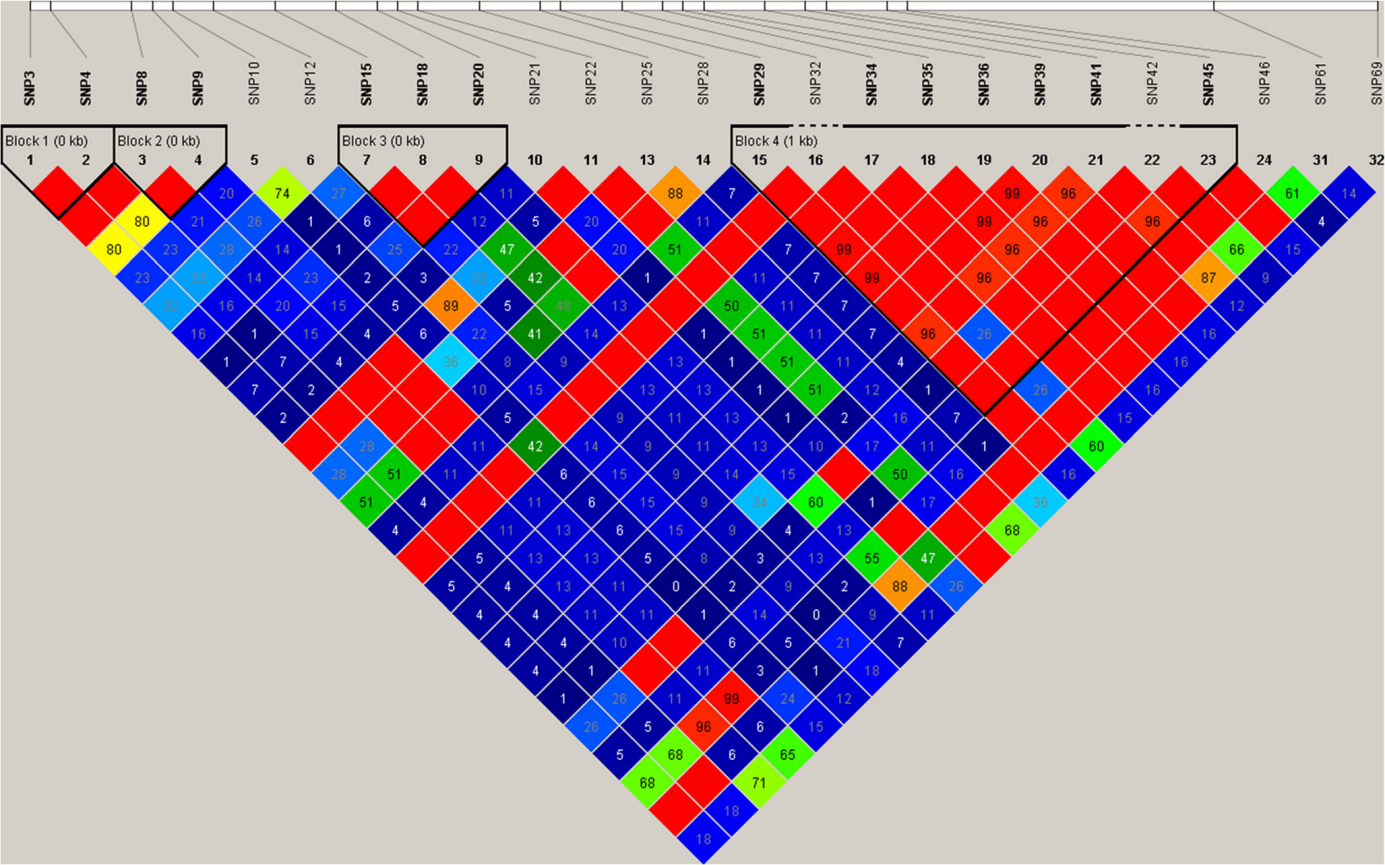
Blocks found in the linkage disequilibrium (LD) analysis on 32 SNPs. LD is indicated by standard color schemes with bright red (very strong: LOD > 2, D′ = 1), light red (LOD > 2, D′ < 1) and blue (LOD < 2, D′ = 1) for intermediate LD, and white (none: LOD < 2, D′ < 1)

We utilized Haploview software for linkage disequilibrium analysis and build haplotypes. Through analyzing all the 32 SNP loci, we detected the existence of 6 blocks (Fig. 1). From the results, we observed the blocks with the powerful association which listed in Table 4. The block which is consisted of SNP15, SNP18, and SNP20 carried the genotype “GGA” which significantly increased the difference of trait. And we found that the other block which consisted of SNP29, SNP32, SNP34, SNP35, SNP36, SNP39, SNP41, SNP42, SNP45, and SNP46 is the genotype “GACGAGTCCG” that indicated the negative difference between the sheep. But with the genotypes “AATCTACTTA” and “AATCTACCTG”, the difference would become wider.

**Table 4:** The built haplotype blocks after linkage disequilibrium analysis in these SNPs

Block	SNPS	Haplotype	Tan_F	Hu_F	OR	95% CI	*p*
HAP1^a^	SNP3;SNP4;SNP8	CAC	100 (0.200)	86 (0.247)	0.7616	0.5488–1.0569	0.1033
		TGC	386 (0.772)	218 (0.626)	2.0192	1.4938–2.7293	0
		TGT	14 (0.028)	44 (0.126)	0.199	0.1073–0.3693	0
HAP2^a^	SNP8;SNP9	CT	486 (0.972)	303 (0.871)	5.1556	2.7825–9.5525	0
		TA	14 (0.028)	44 (0.126)	0.199	0.1073–0.3693	0
HAP3^a^	SNP15;SNP18;SNP20	AGA	297 (0.594)	216 (0.621)	0.8941	0.6753–1.1837	0.4343
		GGA	21 (0.042)	2 (0.006)	7.5846	1.7667–32.5610	*0.0064*^*b*^
		AAA	138 (0.276)	87 (0.250)	1.1436	0.8372–1.5624	0.3991
		GGT	44 (0.088)	43 (0.124)	0.6844	0.4388–1.0676	0.0946
HAP4^a^	SNP21;SNP22	AG	472 (0.944)	320 (0.920)	1.475	0.8572–2.5380	0.1604
		GG	24 (0.048)	28 (0.080)	0.5762	0.3281–1.0122	0.0551
HAP5^a^	SNP22;SNP25;SNP28	GGG	479 (0.958)	337 (0.968)	0.7445	0.3543–1.5647	0.4362
		GGA	8 (0.016)	11 (0.032)	0.4982	0.1983–1.2515	0.1382
HAP6^a^	SNP29;SNP32;SNP34; SNP35;SNP36;SNP39; SNP41;SNP42;SNP45;SNP46	GACGAGCCCG	24 (0.048)	23 (0.066)	0.7125	0.3953–1.2841	0.2593
		GACGAGTCCG	96 (0.192)	105 (0.302)	0.5499	0.3996–0.7568	*0.0002*^*b*^
		AATCTACTTA	32 (0.064)	5 (0.014)	4.6906	1.8090–12.1622	*0.0015*^*b*^
		AATCTACCTG	339 (0.678)	213 (0.612)	1.3345	1.0028–1.7760	*0.0478*^*b*^

^a^HAP: Haplotype block

^b^Shows that the single domain has significant statistical significance (*p* < 0.05)

## Discussion

In view of the outstanding problems such as cultivar recession and quality degradation in Tan sheep, the in-depth and meticulous research on the intrinsic factors and genetic laws affecting the good traits of Tan sheep has plagued the protection of Tan sheep breeds and the healthy development of meat industrial development. We wanted to find out some molecular markers of excellent traits in Tan sheep compared with Hu sheep. Molecular marker–assisted breeding served to carry out targeted breeding of Tan sheep and builds a quality breeding system for Tan sheep. In our research, we randomly selected 32 SNPs and found SNP69 located in CAST; SNP42 and SNP46 located in H-FABP are the most significant sites with positive association with the growth trait. Regardless of the sites whether negative or positive association with the meat quality trait, all of these indicated the differences between Tan sheep and Hu sheep.

Fatty acid–binding proteins (FABPs) can be combined with retinol or retinoic acid–binding protein (RBP or RABP) to compose intracellular lipid–binding proteins, which is an important component of intracellular lipid–binding proteins (ILBPs) and accounts for 1 to 8% of total cellular soluble proteins (Haunerland and Spener 2004). The elementary functions of FABPs (fatty acid–binding proteins) are mainly involved in the storage, transport, and metabolism of fatty acids in animal cells and are closely linked to metabolism and inflammation. H-FABP is a member of the FABPs family. The protein tertiary structure mainly consists of 2 short α-helices and 10 antiparallel β-sheets near the N-terminus whose void center in the β-barrel can bind to the H-FABP ligand. A lot of research has been confirmed that H-FABP genes exists in different species. And found the sheep’s H-FABP gene is located on chromosome 2 (Calvo et al. 2002). The study found that H-FABP gene expression levels have an influence on the final differentiation state of cells after mitosis, which mainly depends on fatty acid metabolism. H-FABP has a strong affinity for long-chain fatty acids and has the function of transporting long-chain fatty acids and balancing the metabolism of fatty acids. The stronger the fatty acid metabolism, the higher the expression level of H-FABP gene. H-FABP gene is indispensable for animal physiological regulation, such as long-chain fatty acid uptake, oxidation, fuel selection, and energy metabolism balance (Schaap et al. 1999). The content of IMF is directly related to the flavor of meat. Some researchers found that the increase in IMF content significantly affected the marbling score (*p* < 0.05) (Cabling et al. 2015). *H-FABP* is considered as one of the major genes that affect the IMF content. The study of Arnyasi shows different genotypes of pigs; H-FABP have a significant effect on IMF content (Arnyasi et al. 2006). The study on Hu sheep showed that the H-FABP mRNA expression level was positively related to IMF content and was positively effective on IMF accumulation in the early development stage (Chengli et al. 2008). Previous studies have shown that the *H-FABP* gene may be an important gene that affects slaughter traits and controls meat quality (Huang et al. 2006). In our results, we found that the SNPs located in *H-FABP* may cause the level of H-FABP expression more than four times higher in Tan sheep than Hu sheep which means Tan sheep meat quality is four times more delicious than Hu sheep. It may be one of the reasons why people prefer to eat Tan sheep meat than Hu sheep.

Calpastatin (CAST) is an important intracellular protein with a molecular mass of approximately 120 ku that specifically inhibits calpain activity (Murachi 1989). Freking et al. mapped the sheep’s CAST gene on chromosome 5 (Freking et al. 1998). *CAST* plays an important role in muscle formation, degradation, and the tenderization process after slaughter (Ranjbari et al. 2012). After the animals are slaughtered, the ATP (adenosine triphosphate) gradually degrades which in turn enhances the ability of the cells to release calcium ions, so that the calcium-ion concentration in the sarcoplasm finally reaches the level of 10^−6^ M, eventually activating the CAST activity. Active calpains first decompose filaggrin-, actin-, tropomyosin-, and connexin-binding subunits. Then, the interaction force between the adjacent sarcomere and the Z disk decreases, and finally, it breaks into small segments of multiple sarcomere, and the binding force between myosin and actin in the actomyosin begins to decrease significantly. When the lysosome breaks down, the released tissue protein interacts with calpain to promote tenderization of meat (Kretchmar et al. 1994). Ropka et al. researched a number of pig breeds in Poland and found that the CAST gene not only has an effect on the pH, hardness, and toughness of pork, but also significantly affects the skeletal hydraulic and intramuscular fat content of pork (Ropka-Molik et al. 2014). The results of Casas et al. showed that the CC genotype of the CAST gene was significantly associated with iron and heme iron in beef (Casas et al. 2014). The study of Calvo et al. indicated that the exon 7 of the cattle CAST gene (g.98535683A>G) is related to the tenderness of beef (Calvo et al. 2014). Our study showed a clear correlation between the *CAST* SNP69 and the difference in meat quality traits of the different species sheep (*P* < 0.05), indicating that the SNPs in *CAST* can be used as a molecular marker to promote the meat quality of Tan sheep. However, the same polymorphic site may have different effects on meat quality in different parts; the results still need further verification.

In addition, we also found that several SNPs in *GH* and *GHR* have statistical significance. As the receptor and ligand exert biological functions, their importance is unquestionable. As important regulators of mammalian growth and metabolism, GH and GHR can regulate the expression of many genes by means of endocrine and nervous system conduction pathways to stimulate tissue metabolism, thereby promoting cell proliferation, bone growth, and protein synthesis (Komisarek et al. 2011). A lot of research proves GH and GHR may be the major genes affecting the important economic traits of livestock or the genes linked to the main gene.

## Conclusion

In the research, we explored the different association between Tan sheep and Hu sheep with growth and meat quality gene, and found that several SNPs in *H-FABP*, *CAST*, *GH*, and *GHR* exist significantly in correlation between the two kinds of sheep. The SNP69, SNP42, and SNP46 with significant statistical difference may be used as a molecular marker for breeding of Tan sheep and had certain effects on the early growth and development of Tan sheep. Our study is only a preliminary study and exist some limitations: First, the sample size is smaller. Second, the research level is relatively junior. It is necessary to further study the function of SNPs and genes in depth by combining protein level research and bioinformatics techniques.

## Electronic supplementary material

ESM 1 (DOC 1371 kb)

ESM 2 (XLSX 15 kb)
