# Supplementary material for: Genetic polymorphism association analysis of SNPs on the species conservation genes of Tan sheep and Hu sheep
Source: Trop Anim Health Prod. 2020 Feb 5;52(3):915–26. doi: 10.1007/s11250-019-02063-1 (PMC7190689; doi:10.1007/s11250-019-02063-1)
Supplement: Supplementary file 1 [file 11250_2019_2063_MOESM1_ESM.doc]

1、GH

（1）. Mutation in Exon 4

Sequence: 5-agagggtgttggatggca-3，5-ggaagggacccaacaatg-3。

:1188~ 1502bp 414bp. T-C mutation occurred in 1406, 

Reference sequence：Sheep GH sequence published in Genebank according to Orian JM.

tggatggca gtggaggatg atggttggtg gtggtggcag gaggtcctcg ggcagaggcc

gacattgcag ggctgccccg agcccgcagc

ctcagcagag tcttcaccaa cagcctggtg tttggcacct cggaccgtgt ctatgagaag

(Red marker is mutatiion site)

 (2). 5' regulatory region, Exon 4, 3'UTR region 

There were polymorphisms in the amplified fragments of GH-P1 primer (5' regulatory region), GH-E4 primer (Exon 4), and GH-P6 primer (3'UTR), and 2 and 1 were detected in these three regions. , 3 SNP loci, a total of 6 SNP loci 

Primer:

GH-P1 

F:AACAGGATGAGTGAGAGGAGGT,

R：CCATCATAGCTGGTGAGCTG

 GH (Accession No.EF077162):For the alignment, the T→C and G→A mutations occurred at the 84th and 85th bp of the amplified fragment of GH-P1 primer (86bp and 87bp of EF077162), respectively. These two SNPs are located in the 5’-UTR regulation area.

gaacaggatg agtgagagga ggttctaaat tatccattag cacaggctgc cagtggtccttgcataaatg tatagagcac acaggtgggg ggaaagggag agagaagaag ccagggtata

GH-P6,

F:GGCAGGAGCTGGAAGATGTC,

R：CCTACTCAGACAATGTGATGCAA

Gtcgccgctt cggggaggcc agctgcgcct tctagttgcc agccatctgt tgttaccccccc(t)cgtgcct tcctagaccc tggaaggtgc cactccagtg cccactgtcc tttcctaataaagcgaggaa attgcatcac attgtctgag taggtgtcat tctattctag ggg

GH-P6 EF077162:1864bp）there is a T base insertion, and a G→T mutation at 257 bp (1887 bp of EF077162), and a C→T mutation occurs at 294 bp (1924 bp of EF077162), and these three SNPs are located in the gene sequence.

3. exon-1 

 Refernencr sequence:：X12546

Primer:

GH-1

5’-CCAGTGGTCCTTGCATAAA-3’

5’-GTTCACATTCAGAAGCCCTA-3’

The GH gene exon-1AA type is identical to the GH gene base sequence published by GenBank (X12546). The AB type is mutated to A at 301 bp and the G mutation is 305 at 305 bp.

2. growth hormone receptor, GHR

- Exon 4

Primer:

GHRE4F: ATTAGGACCArCCATTACC

GHRE4R: TATTTCCATTCCCACCA  

Reference GenBank   NO.AY739710.1，

C-T point mutation at base 62

- GHR Exon-10 

 1、Gen-Bank，AY292283

GHR-10A F Primer：5'-GCCAAAACAATAA-GACTGGGAACC-3'， 

GHR-10A R Primer：5'- GGCTGTAGTG-GTAAGGCTTTCTGTG-3'，

2、GHR seq: DQ062719

GHR-10  

5’-TAAGCGACATTACACCAGC -3’

5’-TTGAGTACGAGGCCCTGT-3’

The GHR gene exon-10 AA is identical to the GHR gene sequence published by GenBank (SEQ ID NO: DQ062719). The AB type is 634 G/A mutation and 805 A/C mutation compared with the AA type. 

3.  Neuropeptide Y, NPY

（1）N10，

F: 5'-GAAAGAATTCAAAGTGGGGA-3'

R: 5'-CCTCTGGTCTTTATGATGCAT-3'

（2）N17

F: 5'-GAAGACTTTTAGTTTTGGCACCCC-3'

R: 5'-CTGAAATCAGGGTCTCGGGGCTAGAT-3'

（3）N20

F: 5'-CTTGGGGCTTTCTTGGGGTGTGGA-3'

R: 5'-CACAAGAGAATGGAGGGAGAAC-3'

（4）N21

F: 5*-CTCCCTCCATTCTCTTGTGAAAT-3'

R: 5'-GGCCACAAAACAAAGAACAGCGTTA-3'

The N10 primer pair amplification region is located on intron 1 of NPY, and A-A/G mutation occurs in the standard sequence of 2253 (302 bp N10 primer pair 40). Missing mutations are 2405-2417 bp deletion TCTTAATAGACCT, and CTTAATAGACCTT deletion at 2432-2444 bp

The N17 primer pair amplification product includes a 47 bp exon sequence of NPY and a 307 bp intron 2 sequence, and the sequencing result showed that the mutation site was located on the  intron 2.

At the 4601 position (the 354 bp N17 primer pair 115 of the amplified product), a C-T transversion occurred.

The N20 primer pair amplification product is completely located in the 2nd intron region of the NPY gene, and the 5560 standard sequence (313bp N20 primer pair 92 of the amplification product) undergoes A-C transversion.

The N21 primer pair amplification product is located in the second intron of the NPY gene, and the A-T conversion occurs in the 5969 standard sequence (343 bp N20 primer pair 207 of the amplification product).

4. Leptin  SNPs

Reference Genbank: AY911719、EF534370

（a） I ntron 2

Primer: EX2

LeptinIN2-F Site: U84247 10~30 bp       5'-CGCAAGGTCCAGGATGACACC- 3'

LeptinIN2-Rsite: AY831682 167~188 bp   5'- GTCTGGGAGGGAGGAGAGTGA- 3'

The 99 bases in intron 2 are mutated from A to G, the 115 bases are mutated from G to A; the 150 bases are mutated from C to T; and the 171 bases are mutated from C to T.。

（b）Exon3

（1）

Leptin-E31-F: EF534370 201~222 bp     5'-TACCAACAGATCCTCGCCAGTC-3'

Leptin-E31-R: EF534370 452~471 bp   5'-CTTCAAGGCTTCAGCACCCA-3'

There are 271 bases mutated from G to A, which converts the encoded Arg to Gln; 316 bases are mutated from C to A, which converts the encoded Pro to Gln; 387 bases are mutated from G to T, making the coding Val turns into Leu.

- 绵羊Leptin基因外显子3的SNPs检测2

Refernece : : EF534370

Primer:

LeptinE32-F 5'-TACCAACAGATCCTCGCCAGTC- 3'；

LeptinE32-R 5'-CTTCAAGGCTTCAGCACCCA-3'

Mutation site：(G271A;C316A;G387T和 G433A）

5. 绵羊 H-FABP 基因的SNPs检测

（a）

- Reference Genbank:：AY157617

- Primer:

H-FP2  

H-FP2 F:GATGAGACCACAGCAGAT

H-FP2 R:CAGGGAAGAGAACAAGAG

H-FP4

H-FP4F:AAGACGAAGTGGACATTTG

H-FP4R:GAGACTCCCTGGTGGTGAT

H-FP6

H-FP6F: GAAATAGTGGACGGGAAAC 

H-FP6R: TGGACTTGGAGAAGGTGAC

H-FP7

H-FP7F:CTGCTGGGAAGTCTGAAGGA

H-FP7R:TGGAATGAAGTAGAAAGGGC

H-FP2 

Site: 981G-A，1041A-C，1019T-C，1058G 插入。

H-FP4

Site：2407T-C

H-FP6 

Site：2957C-T

H-FP7 

Site：3341C-G， 3394T-A

There are 981 (G/A), 1014 (A/C), 1019 (T/C), 1058 (-/G), 2407 (T/C), 2957 (C/T), 3341 (C/G), 8 base mutations such as 3394 (T/A). One of them is a single base insertion/deletion (site P2 (at 1058); three are base transversion (1014, 3341, 3394); 4 is base conversion (transition) ) (981 locations and 1019 locations, 2047 locations, 2957locations)

981G-A，1041A，1019T-C

b. H-FABP

 (GenBank:AY157617

H-Fe2up:AGCTCATGCTCATACCCTTC       

H-Fe2do:CCCTCAAGGTGCAACAAT

H-Fe3up :CTCTACCCTCTTTCCACAGT    

H-Fe3do:TTACGAGTCCTAATACCACC

A base C deletion occurred at position 777. The AA genotype had an A--C mutation at 1013, and the BB genotype had single-base mutations at A, G, G-A and T-C at positions 939, 980 and 1018, respectively.

A G-A mutation occurred at position 2884 and a G-T mutation occurred at position 2926. C-T, C-T, and G-A mutations occurred in the 2A locus, 2956 locus, and 3017 locus, respectively, and the C-T mutation occurred in the 2BB locus in the BB genotype.

6.MSTN

（a）MSTN基因Exon1，Exon2， Exon3的SNPs检测

Reference Genbank: DQ530260.1

Primer:

MSTNP1    F： 5'-AGCAAGAGCCAATCACAGATCC-3'        Exon1       634
MSTNP1   R： 5'-ACAGCAGTCAGCAGAGTCGTT-3'
MSTNP2   F： 5'- ATAGATTGACATGGAGGCGTTC-3'       Exon2          598
MSTNP2 R： 5'- TATTGGGTACAGGGCTACCATC -3'
MSTNP3   F： 5'- GGAGAGTGT TTGGGGATCTATT -3'        Exon3        422 
MSTNP3   R： 5'- ACCCACAGCGATCTACTACCAT -3'

The P1 primer detected three mutation sites in the 5' UTR region. Among them, 216~588 bp is the coding sequence of exon 1, the mutation of A→G at position 127, the mutation of A→G at position 161, and the mutation of C→A at position 175

.

Among the P2 primers, 76~449 bp is the coding sequence of exon 2, the mutation of A→G at position 407 of the P2 product fragment, and the mutation of T→C at position 571.

The P3 primer amplified the sequence of the P3 product fragment of MSTN gene (422 bp), of which 50-422 bp was the mutation of exon 3 in the 53rd C→T of the P3 product fragment, and the mutation of the 76th A→T , mutation at position 88 → A → G, mutation at T→C of the 46th sheep, and transformation mutation at position 329 A → G.

(b). Non-coding area of MSTN

Reference Genbank:DQ530260

3′UTR Primer:

MSTN-3U -F: 5′-TTTGGTATATTTTTACAGTAAGGAC-3′;
          R: 5′-TAAATAGTGTTGCACTTAAGGATTC-3′。
5’UTR Primer

MSTN-5U  F: 5′-CAGCAACAAGCAGCATAA-3′;
           R: 5′-GAGCAAGAGCCAATCACA-3

The corresponding positions of DQ530260 are: 1~1 003 bp→9556~10 558 bp; 1~271 bp→3389~3 659 bp),

As a result, a single base mutation of G→A was found at the 272 bp of the 3′UTR region, and a single base mutation of T→C was observed at the 910 bp.

Sequencing revealed a single base mutation of C→A at the 176 bp of the 5′UTR region and a T→C mutation at its adjacent 179.

7.CAST 

（a）Exon 6

  CAST -E6 F: ATTGCTTTCTACTCCTCAGA

          R: ATACGATTGAGAGACTTAC

A/G base substitution occurred at 56 bp, and A/C base substitution occurred at 184 bp.

.

(b)  3’-UTR 

CAST-S3 5’-CTCTTGGACTTTTGAACTGTA-3’

        5’-CCACGTGAGTATTCTAAGCA-3’ 

CAST-S7 5’-GACTTGGGTGTCTGCTGG-3'

        5’-ACCTGAGGCACCTATTTT-3;

 There are 4 mutation sites in the CAST-S3 amplification product, in which there is a mutation of AG at the 73 position of the amplified product (Fig. 4-3A), and there is a deletion of CA at the 105-106 site (Fig. 4-3B) There is a mutation in GA at position 210 (Fig. 4-3C), and a mutation in GA at position 219 (Fig. 4-3D).

There are 4 mutation sites in the CAST-S7 amplification product, in which there is a deletion of T at the 80th position of the amplified product, a mutation of AG at the 83 site and the deletion of T in the tested individual is found. The mutations in AG occur simultaneously (Fig. 4-4A), there is a mutation in GA at position 158, and a mutation in GT exists at position 163.
